# Distinct media requirements for dopaminergic neuron reprogramming from human glia and fibroblasts

**DOI:** 10.1038/s41598-026-66620-z

**Published:** 2026-08-17

**Authors:** Kerstin Laurin, Janko Kajtez, Jenny Wickham, Malin Parmar, Mette Habekost

**Affiliations:** 1 Developmental and Regenerative Neurobiology, Lund Stem Cell Center, Department of Experimental Medical Science, Faculty of Medicine, Lund University, Lund, Sweden; 2 Novo Nordisk Foundation Center for Stem Cell Medicine (reNEW), Department of Biomedical Sciences, Faculty of Health and Medical Sciences, University of Copenhagen, Copenhagen, Denmark; 3 Laboratory of Stem Cells and Restorative Neurology, Lund Stem Cell Center, Faculty of Medicine, Lund University, Lund, Sweden; 4 Center for Translational Neuromedicine, Faculty of Health and Medical Sciences, University of Copenhagen, Copenhagen, 2200 Denmark; 5 Present Address: Department of Biotechnology and Biomedicine, DTU Bioengineering, Technical University of Denmark, Kgs. Lyngby, 2800 Denmark

**Keywords:** Direct fibroblast to neuron reprogramming, Direct glia to neuron reprogramming, Induced dopaminergic neurons, Media composition, Culture conditions, Cell biology, Neuroscience, Stem cells

## Abstract

Direct reprogramming of somatic cells into induced dopaminergic neurons (iDANs) is a promising strategy for replacing degenerated dopamine neurons in Parkinson’s disease. One current bottleneck for translation of direct neuronal reprogramming is the low reprogramming efficiency and difficulty to acquire enough tyrosine hydroxylase (TH) positive iDANs. Here we systematically refine the culture conditions used for dopaminergic reprogramming of human stem cell-derived glial progenitor cells (GPCs) and adult dermal fibroblasts (DFs). Starting from a standard reprogramming medium containing base medium, growth factors and small molecules, we systematically compared three base media and removed individual small molecules to determine their specific contribution to reprogramming outcome. The GPCs and DFs were reprogrammed using established transcription factor-based strategies combined with *REST* inhibition. We found that the two starting cell types benefited from different culture conditions. For GPCs, B27/Neurobasal medium without small molecules yielded the highest neuronal and TH reprogramming efficiency, whereas DFs yielded the highest efficiencies in N2/DMEM/Neurobasal containing small molecules. Further characterization of the GPC-derived iDANs showed that these cells were functional, expressed key dopaminergic markers, and released dopamine upon stimulation. Together, these findings demonstrate that media composition is a key parameter influencing dopaminergic reprogramming efficiency across starting cell types in vitro.

**Supplementary Information:**

The online version contains supplementary material available at 10.1038/s41598-026-66620-z.

## Introduction

Direct reprogramming of somatic cells into induced neurons (iNs) allows rapid generation of post-mitotic neurons without passing through a pluripotent state. Early studies demonstrated that glial cells could generate neurons after forced expression of developmental transcription factors^1^. Later work refined this approach across multiple somatic cell types by combining transcription factors, microRNAs, and knockdown-based strategies to establish direct conversion of somatic cells into different subtype neurons^2–8^. Several of these early studies showed that adding small molecules modulating developmental pathways could further enhance the conversion efficiency of fibroblasts and astrocytes to iNs, such as inhibiting TGFβ and BMP signaling, modulating Wnt activity, and increasing cAMP signaling in addition to providing neurotrophic support^9–12^. In parallel, it was also shown that small molecules alone could reprogram fibroblasts to iNs^13^. Following these studies, the addition of small molecules to the reprogramming media has become standard practice.

As the field has advanced, direct neuronal reprogramming is now considered not only a powerful in vitro tool for disease modelling but also a promising regenerative strategy for replacing lost neurons^14^. In this context, iNs generated from accessible somatic cell types, such as dermal fibroblasts (DFs), may provide a source of patient-specific neurons for transplantation-based approaches^15^. In parallel, the ability to convert glial cells into neurons opens up opportunities for in situ reprogramming as a therapeutic approach to neurodegenerative diseases. Parkinson’s disease, characterized by the progressive degeneration of midbrain dopaminergic (DA) neurons, represents a major target for regenerative medicine and has emerged as a key model for developing strategies to generate DA neurons either through transplantation of reprogrammed fibroblasts or by direct in situ conversion of resident glial cells^16,17^.

We and others have previously shown that human glia and fibroblasts can be converted into DA neurons in vitro^15,18–24^. However, while overall neuronal conversion is efficient, the yield of induced DA neurons (iDANs), assessed by tyrosine hydroxylase (TH) expression, remains relatively low. This can in part be due to insufficient delivery of DA reprogramming factors, but also suggests that current extracellular signaling conditions may not sufficiently support or stabilize DA identity. Notably, several small molecules routinely used in direct reprogramming protocols are used as generic pro-neural cues. For example, Wnt activation via CHIR99021 is commonly used to support neuronal conversion using the same concentration regardless of desired neuronal subtype^9,18,25^.

Improvements in transcriptional reprogramming, including strategies that remove intrinsic barriers to neuronal identity such as REST^26,27^, may reduce the need for complex extracellular signaling to boost conversion efficiency. As a result, media compositions from earlier protocols with lower yields may no longer provide an optimal balance between overall neuronal conversion and subtype specificity. In this study, we therefore evaluate the media requirements of our standard reprogramming medium with widely used small molecules, as those applied in our glial progenitor cell (GPC) and fibroblast–based reprogramming protocols^28,29^. By systematically removing small molecules and testing alternative basal media, we identified optimized culture conditions that support robust neuronal conversion while improving iDAN yield. To assess the generality of these requirements, we performed reprogramming in both human GPCs and DFs, enabling comparison of media composition requirements across starting cell types.

## Results

### Direct reprogramming of human GPCs and DFs to iDANs in standard culture conditions

**Fig. 1:**
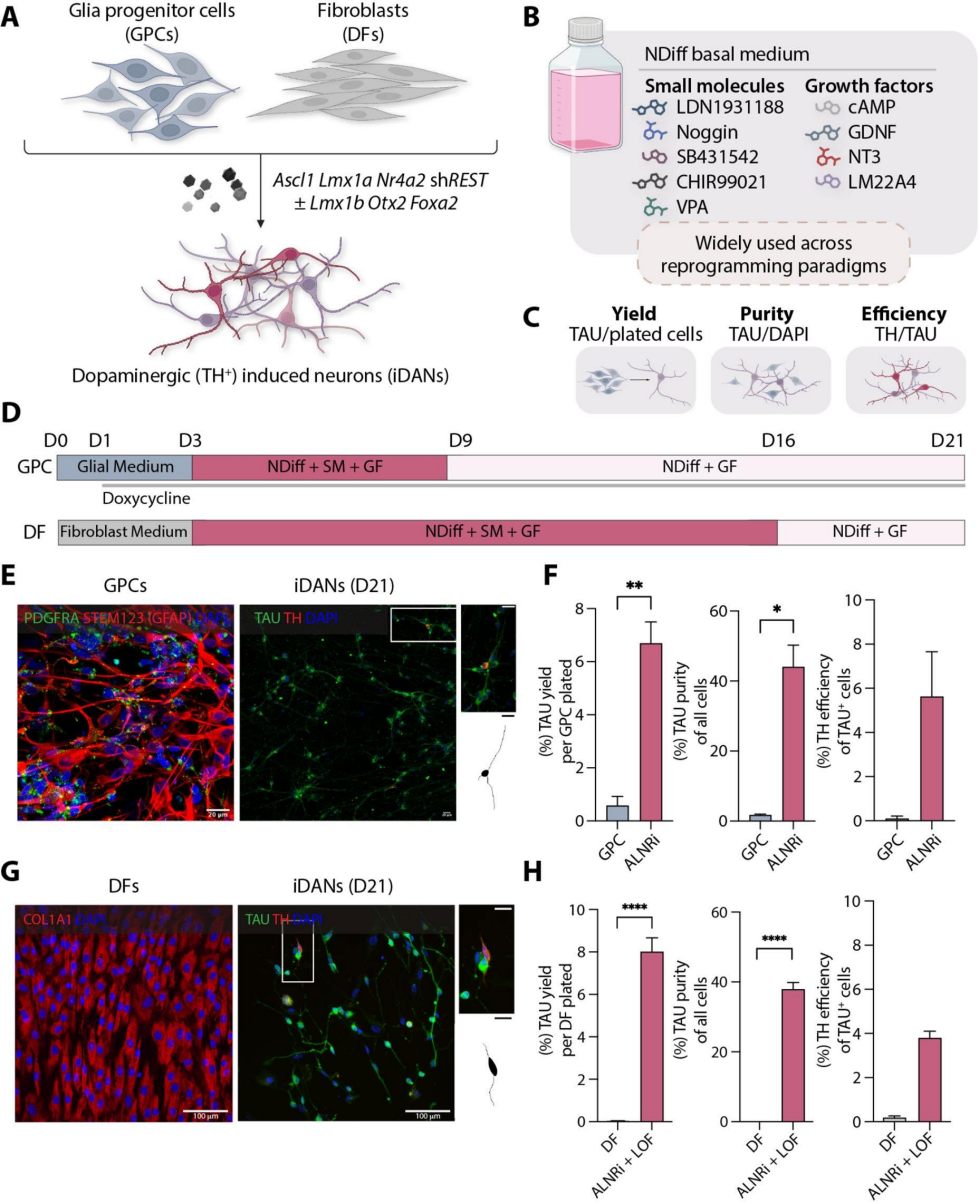
Direct reprogramming of GPCs and DFs into iDANs using NDiff medium and small molecules. (**A**) Schematic of the generation of induced dopaminergic neurons (iDANs) from human stem cell-derived glia progenitor cells (GPCs) and dermal fibroblasts (DFs). (**B**) Formulation of the early NDiff reprogramming medium used in current protocols. (**C**) Illustration describing the definitions of TAU yield, TAU purity, and TH efficiency. (**D**) Timelines showing the media composition at early and late stages of the protocols for reprogramming GPCs and DFs using NDiff, small molecules (SM) and growth factors (GF). (**E**) Representative immunostaining of GPCs for the glial markers PDGFRα and STEM123 (GFAP) before reprogramming and of iDANs reprogrammed for 21 days (+ *Ascl1*,* Nr4a2*,* Lmx1a* and knockdown of *REST* (ALNRi) in NDiff medium) for the neuronal marker TAU and the dopaminergic marker TH with reconstruction of a TH^+^ iDAN. Nucleus stained for DAPI, scale bar 20 μm. (**F**) Quantifications of TAU yield, TAU purity and TH efficiency of GPCs and reprogrammed GPCs (ALNRi (NDiff)). Mean ± SEM; Welch’s t test comparing ALNRi to GPCs; ** *P* = 0.008 and * *P* = 0.02. *N* = 3 replicates (GPC batches) from RC17. (**G**) Representative immunostaining of DFs for fibroblast marker COL1A1 before reprogramming, and of iDANs reprogrammed for 21 days (+ *Ascl1*,* Nr4a2*,* Lmx1a*,* Lmx1b*,* Otx2*,* FoxA2* and *REST* knockdown (ALNRi + LOF) with NDiff medium) for TAU and TH. Nucleus stained for DAPI, scale bar 100 μm. Reconstruction of a TH^+^ iDAN, scale bar 20 μm. (**H**) Quantifications of TAU yield, TAU purity and TH efficiency of DFs and reprogrammed DFs (ALNRi + LOF (NDiff)). Mean ± SEM; Welch’s t test comparing ALNRi + LOF to DFs; **** *P* < 0.0001. *N* = 4 replicates (DFs lines).

We previously showed that direct reprogramming of GPCs into iDANs can be achieved by overexpression of *Ascl1*, *Lmx1A*, *Nr4a2* and knockdown of *REST* using shRNAs (ALNRi) while the reprogramming of human adult DFs requires additional factors such as *Lmx1B*, *Otx2* and *FoxA2* (ALNRi + LOF) (Fig. 1A)^18,20^. In both protocols, a defined set of small molecules that modulate developmental pathways and neurotrophic factors is added to the base medium (“NDiff 227 - neural differentiation medium” from TakaraBio) to improve neuronal reprogramming efficiency^18,20^(Fig. 1B). During conversion, small molecules are included in the medium between days 3–9 for GPCs to aid the early stages of reprogramming (Fig. 1D top). For DFs they are added between days 3–16 (Fig. 1D bottom).

To comparatively assess the reprogramming outcome under these conditions in our standard medium, we converted GPCs and DFs and quantified the TAU yield, TAU purity and TH efficiency after 21 days. During this process, the cells shifted from expressing the glial markers PDGFRα and GFAP to expressing neuronal markers such as TAU and the DA marker TH (Fig. 1E). This resulted in a TAU yield (defined as TAU positive (TAU^+^) cells over number of plated cells) of 6.67 ± 0.80 SEM %, TAU purity (defined as TAU^+^ cells over total cells counted with DAPI) of 44.06 ± 6.22 SEM %, and a TH efficiency (defined as number of TH positive (TH^+^) cells over TAU^+^ cells) of 5.63 ± 2.03 SEM % (Fig. 1C and F and Table S1). Similarly, the reprogramming of DFs into iDANs using ALNRi + LOF over 21 days with presence of the fibroblast marker COL1A1 at day 0 and expression of TAU and TH at day 21 (Fig. 1G), and resulted in a TAU yield of 8.02 ± 0.66 SEM %, TAU purity of 37.96 ± 1.91 SEM %, and a TH efficiency of 3.81 ± 0.3 SEM % (Fig. 1H and Table S1). Thus, these protocols performed in our standard NDiff medium support successful conversion of GPCs and DFs to induced neurons but are limited by a relatively low yield, purity, and efficiency of cells positive for the DA marker TH.

### Testing alternative base media and small molecule combinations for DA reprogramming of GPCs

**Fig. 2:**
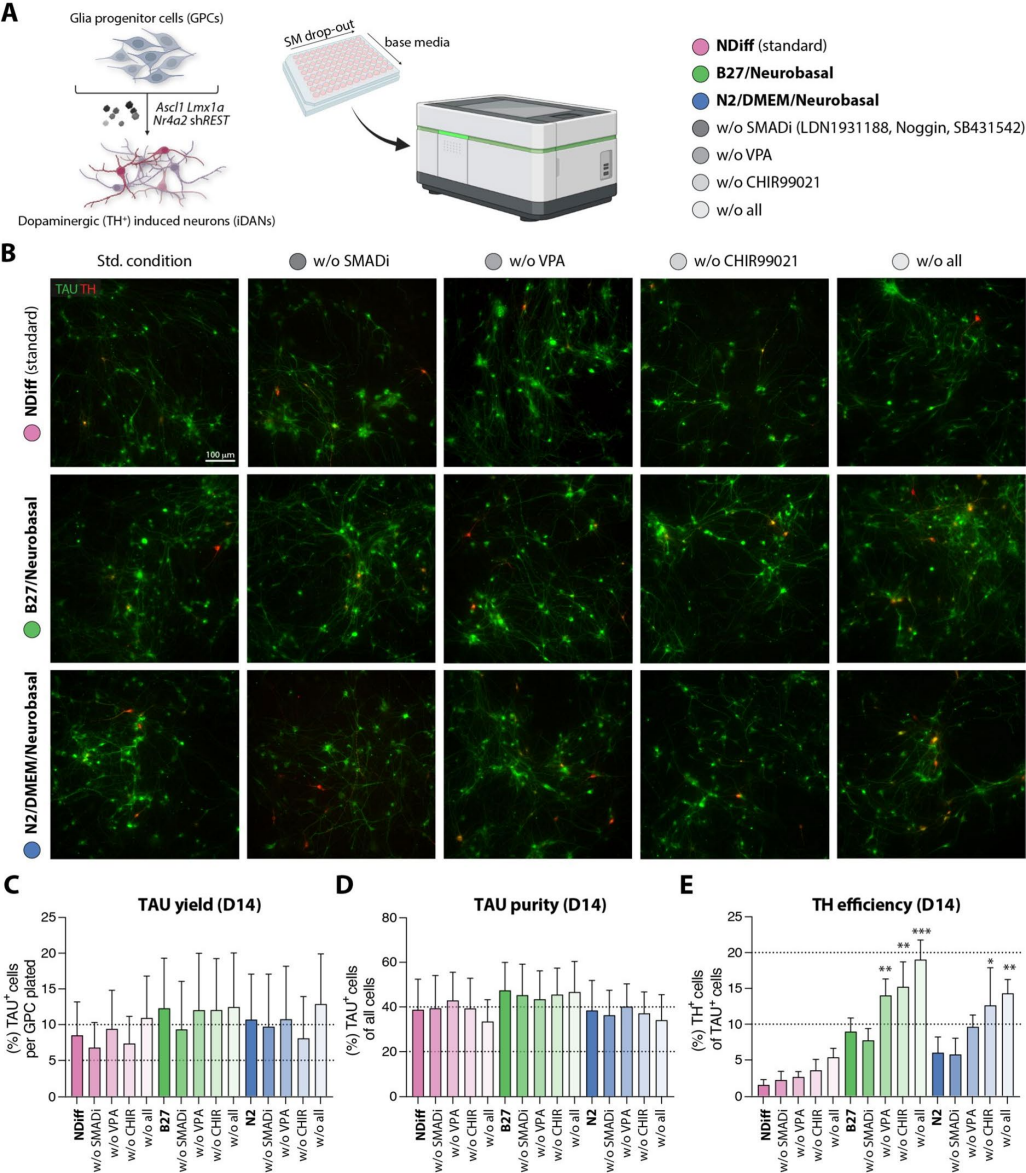
GPC reprogramming screen across base media and small molecule combinations. (**A**) Schematic illustration of experimental layout and color-coding of base media and small molecule combinations tested. (**B**) Representative images of reprogrammed GPCs expressing TAU and TH for each condition tested. Scale bar 100 μm. (**C**-**E**) Quantifications of (**C**) TAU yield, (**D**) TAU purity, (**E**) TH efficiency, for the three base media and the five small molecule combinations tested at day 14 of reprogramming; mean ± SEM; one-way ANOVA with Dunnett’s post hoc test (multiple comparisons) comparing each condition to NDiff (standard).; * *P* < 0.05, ** *P* < 0.01, *** *P* < 0.0001; (*N* = 3 replicates (GPC batches) from 2 ESC lines).

We first tested the influence of media composition on GPC reprogramming outcomes, by testing three base medium formulations: NDiff, B27/Neurobasal, N2/DMEM/Neurobasal in combination with five small molecule conditions: the full small molecule cocktail (standard condition), without SMAD inhibitors (i.e., LDN1931188, Noggin, SB431542), without VPA, without CHIR99021, and without all small molecules (Fig. 2A). This allowed us to compare the standard reprogramming condition with two alternative media and to assess the contribution of the individual small molecules by systematic removal. The small molecules were included only during day 3–9 to match our standard reprogramming protocol (Fig. 1D top). In this screen, the GPCs were reprogrammed for 14 days after which TAU yield, TAU purity and TH efficiency were quantified for each condition (Fig. 2B-E and Table S2). No significant differences in TAU yield or purity were observed between the different conditions (Fig. 2C and D). In contrast, TH efficiency was significantly increased in several conditions and most prominently in conditions without CHIR99021 and without all small molecules (Fig. 2E). Removal of CHIR99021 increased TH efficiency in B27/Neurobasal to 15.22 ± 3.50 SEM % and N2/DMEM/Neurobasal to 12.60 ± 5.26 SEM %, and removal of all molecules further increased the efficiency in B27/Neurobasal to 19.03 ± 2.74 SEM % and N2/DMEM/Neurobasal to 14.30 ± 1.96 SEM % compared to standard NDiff condition 1.53 ± 0.74 SEM %. The NDiff conditions performed poorly overall compared to B27/Neurobasal and N2/DMEM/Neurobasal (Fig. 2B-E). As successful reprogramming requires co-delivery and expression of 4 independent vectors, we assessed the protein expression of Ascl1, Nurr1 and Lmx1a in GPCs reprogrammed in B27/Neurobasal without small molecules (*B27 w/o all*) to estimate co-delivery of the vectors. At day 14, 45.8 ± 2.86 SEM % expressed ASCL1, 47.33 ± 2.57 SEM % expressed NURR1, 49.11 ± 1.99 SEM % expressed LMX1A while 32.33 ± 2.13 SEM % co-expressed all three (Fig. S1A-B). Based on the overall neuronal yields and purities (Fig. 2C-E and Table S2), the *B27 w/o all* was selected for further experiments.

### Functional and phenotypic characterization of GPCs reprogrammed in B27/Neurobasal without small molecules

**Fig. 3:**
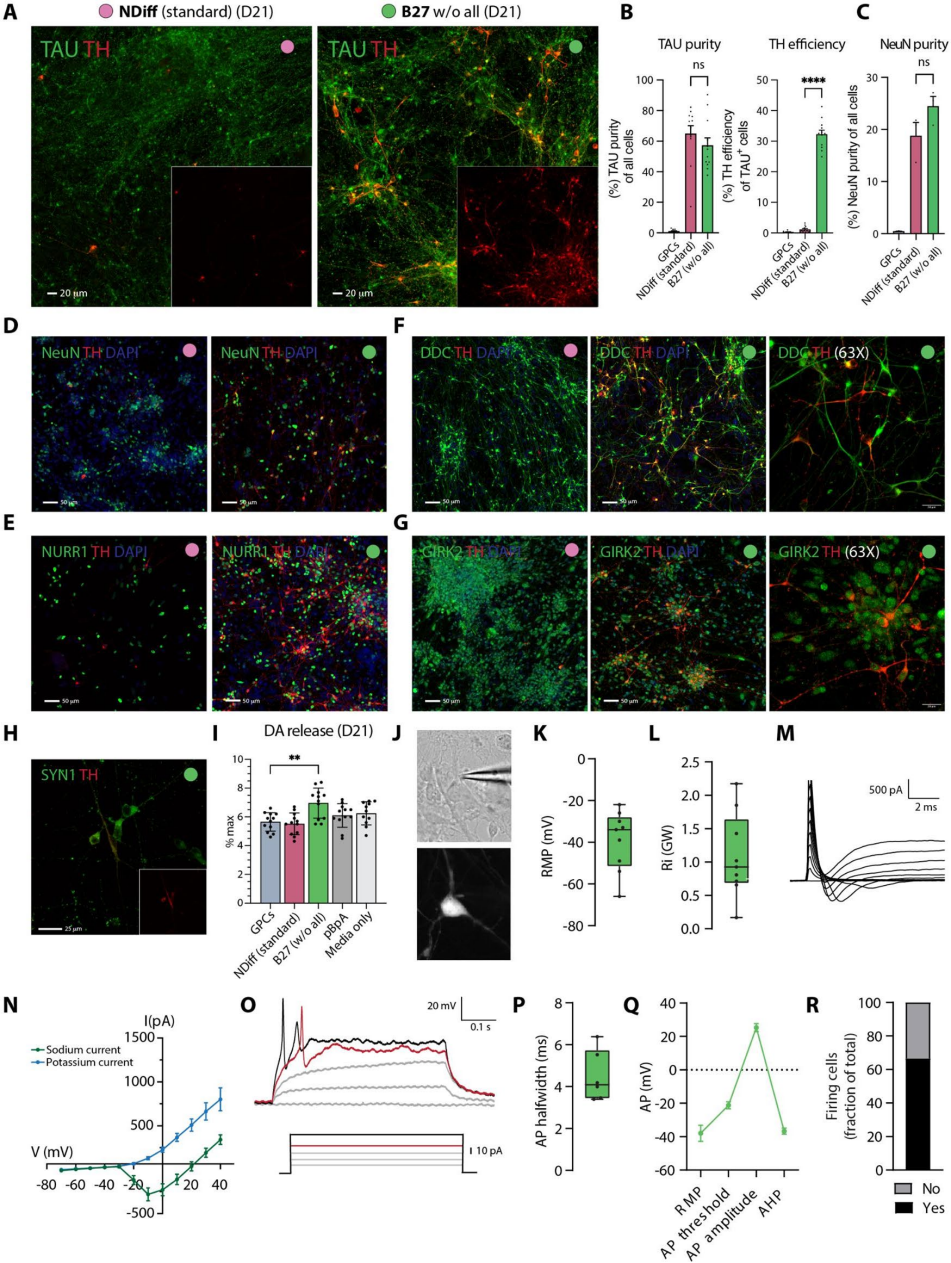
Dopaminergic and functional characterization of GPCs reprogrammed in *B27 w/o all* medium. (**A**) Immunocytochemistry (ICC) of induced dopaminergic neurons (iDANs) at day 21 (D21) showing TAU and TH expression in standard medium (NDiff; to the left) and in *B27 w/o all* (to the right). Scale bar 20 μm. (**B**) Quantifications of TAU purity and TH efficiency of iDANs at D21 reprogrammed in NDiff and *B27 w/o all* medium with non-reprogrammed GPCs as control; mean ± SEM; Welch’s t test comparing NDiff to *B27 w/o all*; **** *P* < 0.0001. *N* = 4 replicates (GPC batches) with 2–6 technical (individual conversions) replicates from 2 ESC lines and 1 iPSC line. (**C**) Quantifications of NeuN^+^ cells in NDiff and *B27 w/o all* medium; mean ± SEM; Welch’s t test. *N* = 3 replicates (GPC batches) from 1 ESC line (**D**) ICC of NeuN, TH and DAPI. Scale bar 50 μm. Related to (**C**). (**E**-**G**) ICC of the dopaminergic markers NURR1 (**E**), DDC, and GIRK2 (**G**) co-expressed with TH and DAPI. Scale bar 50 μm. High magnification representation of DDC and GIRK2 co-localization with TH in (**F**) and (**G**). Scale bar 20 μm. (**H**) ICC of synapsin (SYN1) and TH co-localization in *B27 w/o all* medium derived iDANs at D14. Scale bar 25 μm. I) Quantification of dopamine (DA) released from control GPCs, ALNRi reprogramming in standard NDiff or in *B27 w/o all*, non-DA reprogramming (using pB.pA) in *B27 w/o all* and non-transduced GPCs in *B27 w/o all*. Mean ± sd; One-way ANOVA with Dunnett’s multiple comparison test comparing each condition to control GPCs; ** *P* = 0.0011. *N* = 3 replicates (GPC batches) each with 4 technical replicates from 1 ESC line (RC17) and 1 iPSC line. J) Representative images of a recorded cell during whole-cell patch clamp. An individual GFP reporter was added on top of ALNRi to mark transduced cells. K) Resting membrane potential (RMP) of recorded cells (*N* = 6). L) Input resistance (Ri) of recorded cells. (**M**) Representative trace of inward sodium and outward potassium currents evoked by depolarizing voltage steps. (**N**) Current-Voltage (I–V) relationship for inward sodium (green) and outward potassium (blue); mean ± SEM. (**O**) Representative traces of evoked action potentials (AP). (**P**) AP halfwidth across recorded cells. Q) AP properties of recorded cells; RMP, AP threshold, AP amplitude and afterhyperpolarization (AHP); mean ± SEM. R) Graph showing recorded cells firing at least one AP (Yes) versus non-firing cells (No).

We next sought to characterize the iDANs generated in *B27 w/o all* further by assessing their functional properties and expression of additional DA markers apart from TH. At day 21 of reprogramming an increase of TH^+^ neurons was observed (Fig. 3A), and quantifications revealed that 32.26 ± 1.35 SEM % of TAU^+^ cells expressed TH after reprogramming in *B27 w/o all* compared to only 1.1 ± 0.29 SEM % in standard NDiff condition (Fig. 3B and Table S3). No significant differences in the TAU purity (Fig. 3B) or in the expression of neuronal marker NeuN were observed indicating that *B27 w/o all* did not affect the overall neuronal conversion but rather the specification towards the DA subtype (Fig. 3C and D and Table S4). In support of this, these TH^+^ cells were found to co-express key DA markers such as DOPA decarboxylase (DDC), GIRK2 and NURR1, and more double-positive cells were observed in the *B27 w/o all* condition than in the NDiff condition, consistent with the higher number of TH^+^ cells (Fig. 3E-G). TH^+^cells generated in *B27 w/o all* medium also expressed the more mature neuronal marker Synapsin I after 14 days of reprogramming (Fig. 3H). We therefore next sought to functionally characterize the iDANs generated in *B27 w/o all* by assessing their ability to release DA after KCl stimulation. In this analysis, DA release was detected in supernatant collected after depolarization using GRAB_DA2M_ sniffer cells, which was observed only in the *B27 w/o all* condition in combination with DA reprogramming factor (ALNRi). Neither the NDiff condition or non-DA reprogramming (pB.pA vector containing *Ascl1*, *Brn2* and sh*REST*^26^ in *B27 w/o all* or medium alone released detectable levels of DA after stimulation with KCl at 21 days (Fig. 3I and Table S3).

Whole-cell patch clamp recordings were performed on GFP-positive cells by co-delivering a GFP reporter with the reprogramming cocktail (Fig. 3J). Because GFP labelled transduced cells rather than DA neurons specifically, the identity of the recorded cells could not be determined prior to recording. Cells reprogrammed in the *B27 w/o all* for 35 days showed that the resting membrane potential (RMP) was − 39.22 ± 4.79 mV (Fig. 3K) and input resistance (Ri) was 1.083 ± 0.210 GΩ (Fig. 3L). Cells displayed inward sodium and outward potassium currents (Fig. 3M-N). Out of 9 recorded cells, 6 showed abilities to generate action potentials (AP) following depolarization with RMP, AP threshold, AP amplitude, afterhyperpolarization (AHP) and AP halfwidth recorded (Fig. 3O-R and Table S5). These data show that the cells reprogrammed in *B27 w/o all* had a depolarized resting state and that a proportion of recorded cells generated APs. Such electrophysiological properties are comparable to reprogrammed GPCs with the standard NDiff^19,20^.

**Fig. 4:**
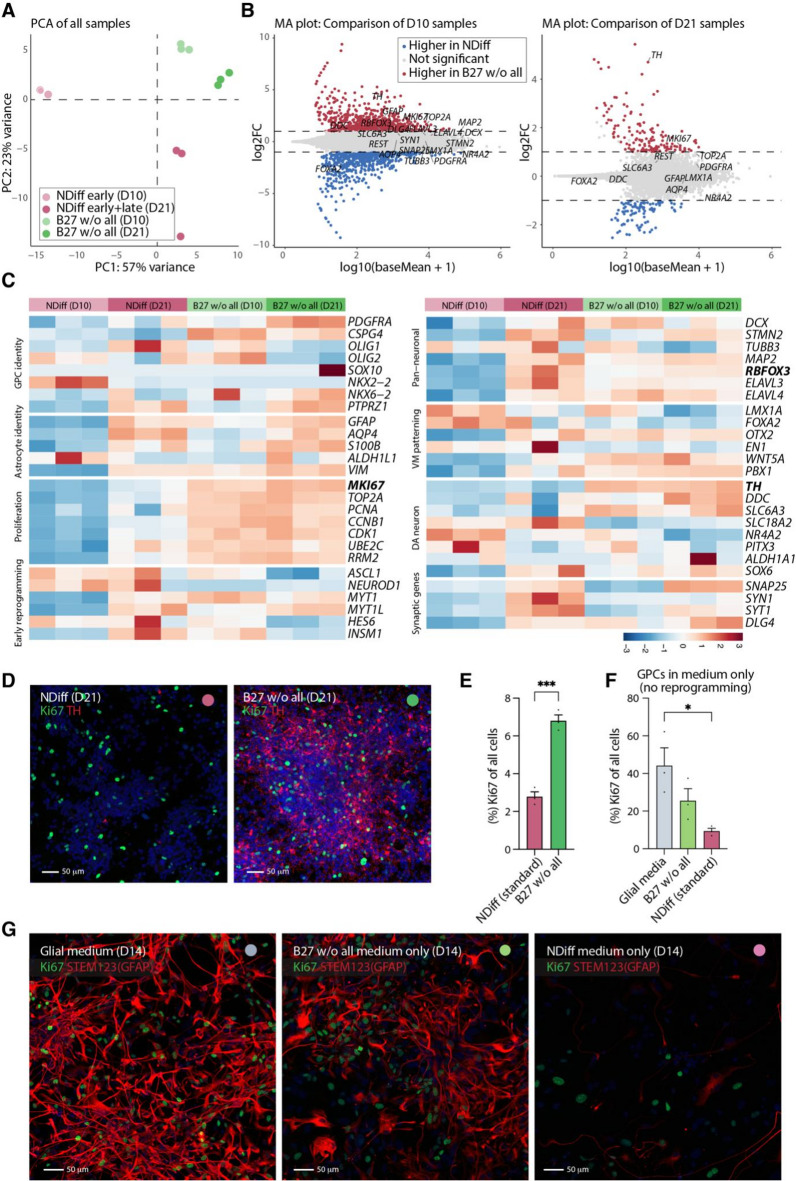
Characterization of gene expression profiles and proliferation. (**A**) Principal component analysis (PCA) of bulk RNA-seq samples reprogrammed in standard NDiff medium or in *B27 w/o all* medium. RNA was harvested at day 10 (D10) or D21 timepoints (*N* = 3 technical (individual conversions) replicates using 1 GPC batch from 1 ESC line (RC17). (**B**) MA plots for D10 and D21 samples of differentially expressed genes (DEG) between NDiff vs. *B27 w/o all*. Up-regulated DEG in *B27 w/o all* with log_2_ fold change (FC) > 1 shown in red and DEG up-regulated in NDiff shown in blue. (**C**) Heatmap ln(TPM + 1) representation of expression of selected genes for the two media at D10 and D21. (**D**) ICC images of TH and Ki67 expression at D21 in the two media conditions. Scale bar 50 μm. (**E**) Quantification of Ki67 positive (Ki67^+^) cells in standard NDiff and *B27 w/o all* medium; mean ± SEM; Welch’s t test; *** *P* = 0.0007. *N* = 3 technical (individual conversions) replicates using 1 GPC batch from 1 ESC line (RC17). (**F**) Quantification of Ki67^+^ cells in glial medium, *B27 w/o all* medium and standard NDiff medium; mean ± SEM; one-way ANOVA with Tukey’s multiple comparisons test comparing all conditions to each other, * *P* = 0.0231; *N* = 3 replicates (GPC batches) from 2 ESC cell lines and 1 iPSC line. (**G**) ICC images of untransduced GPCs expressing Ki67 and GFAP (STEM123) in glial medium, *B27 w/o all* medium and standard NDiff medium. Nuclear counterstain with DAPI. Scale bar 50 μm.

To further explore the molecular differences between iDANs generated from GPCs in the standard NDiff medium and the *B27 w/o all* medium we performed bulk RNA-seq and differential gene expression (DEG) analysis between the two conditions at day 10 and 21 to assess differences at the point where small molecules are removed from the standard NDiff medium (day 10) and when iDANs have started to form (day 21) (Fig. 1D top). Principal component analysis (PCA) showed differences between the media conditions and timepoints (Fig. 4A). In particular, the NDiff late (D21) and *B27 w/o all* (both timepoints) separated from NDiff early (D10) along PC1 (57% variance explained), while NDiff late (D21) also separated along PC2 (23%) (Fig. 4A). DEG analysis revealed differential expression of 1,417 genes (P adjusted < 0.05 and absolute log2 fold change > 1) between the two media conditions at day 10 and 237 genes (P adjusted < 0.05 and absolute log2 fold change > 1) at day 21 with *TH* being one of the top upregulated genes in *B27 w/o all* at both timepoints (Fig. 4B). Gene Ontology analysis on these genes revealed enriched cell-cycle and chromosome segregation processes in *B27 w/o* all samples at D10, whereas NDiff cultures showed enrichment of ribosomal and cell fate specification-related processes (Supplementary Fig. S2A). At D21, the *B27 w/o all* cultures were still enriched for proliferative and extracellular matrix-associated signatures (Supplementary Fig. S2B).

We next plotted the gene expression of selected genes involved in GPC/astrocyte/pan-neuronal/DA neuron identities, proliferation, ventral midbrain (VM; the region where midbrain DA neurons are generated) patterning, and synaptic genes as a heatmap (Fig. 4C). Interestingly, NDiff (D10) samples showed higher expression levels in genes related to VM patterning such as *LMX1A* and *FOXA2* and lower expression in pan-neuronal genes, indicating that this medium composition supports specification of midbrain cells (Fig. 4C). However, the expression of DA genes including *TH*,* DDC* (Dopa Decarboxylase) and *SLC6A3* (DAT) were higher in *B27 w/o all* samples at D10 and D21 (Fig. 4C). In addition, these samples also showed higher expression of genes related to astrocyte identity, GPC identity and proliferation and slightly lower expression of pan-neuronal genes (Fig. 4C). We therefore next assessed the proportion of proliferative cells (Ki67^+^) in the cultures and observed significantly higher proportion of Ki67^+^ cells in *B27 w/o all* medium with 6.8 ± 0.31 SEM % compared to the 2.78 ± 0.26 SEM % observed in the NDiff medium (Fig. 4D-E and Table S6). The Ki67^+^ cells did not co-express TH (Fig. 4D) suggesting that *B27 w/o all* supports continued proliferation of unconverted/untransduced cells. To further explore this, we cultured untransduced GPCs in their normal culturing medium or in *B27 w/o all* and standard NDiff for 14 days and assessed proliferation of GPCs in the different media. We observed higher proportion of Ki67^+^ cells in *B27 w/o all* 25.51 ± 9.45 SEM % compared to standard NDiff 9.34 ± 1.49 SEM % (Fig. F-G and Table S7) together with a higher proportion of cells positive for glial marker GFAP in the *B27 w/o all* compared to standard NDiff. These results indicate that the *B27 w/o all* supports proliferation of unconverted GPCs.

### Analyzing media requirements for iDAN reprogramming of DFs

**Fig. 5:**
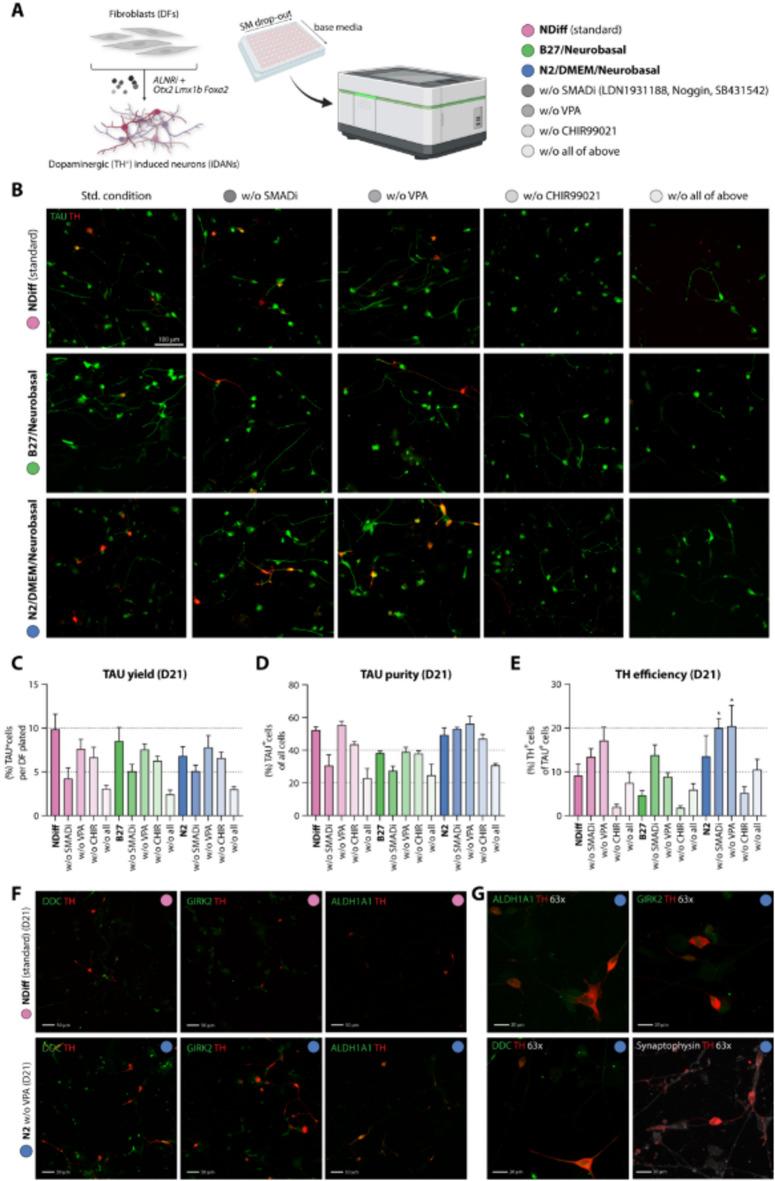
Base media and small molecules screen for reprogramming of DFs. (**A**) Schematic illustration of experimental setup for base media and small molecule screen reprogramming DFs with ALNRi + LOF. (**B**) Representative ICC images of the three base media and five small molecule combinations showing TAU and TH expression. Scale bar 100 μm. (**C**-**E**) Quantifications of (**C**) TAU yield, (**D**) TAU purity, (**E**) TH efficiency, for the 15 conditions tested after 21 days of reprogramming; mean ± SEM; one-way ANOVA with Dunnett’s post hoc test (multiple comparisons) comparing each condition to standard NDiff; * *P* < 0.05; *N* = 3 replicates (DFs lines). (**F**) ICC of dopaminergic markers DDC, GIRK2 and ALDH1A1 co-expressed with TH in standard NDiff and *N2 w/o VPA* medium. Scale bar 50 μm. (**G**) High magnification of ICC showing co-localization of TH with ALDH1A1, GIRK2, DDC and Synaptophysin in *N2 w/o VPA* medium. Scale bar 20 μm.

Having found that the base media and small molecules combinations significantly influenced the reprogramming of GPCs to iDANs, we next asked whether similar influence is observed when reprogramming other starting cell types such as DFs. We therefore performed the same screening during fibroblast to iDAN reprogramming using the established factor combination, ALNRi + LOF^18^, and performed quantifications of TAU yield, TAU purity and TH efficiency as readouts (Fig. 5A). The same three base media and five small molecule combinations were tested, and the DFs were reprogrammed for 21 days (Fig. 5B-E and Table S8). In contrast to reprogramming GPCs, the removal of all small molecules markedly decreased the TAU yield and purity regardless of the base media used (TAU yield; *NDiff w/o all* 3.08 ± 1.15 SEM %, *B27 w/o all* 2.44 ± 0.5 SEM %, *N2 w/o all* 3.01 ± 0.32 SEM %, TAU purity; *NDiff w/o all* 22.87 ± 5.92 SEM %, *B27 w/o all* 24.69 ± 6.78 SEM %, *N2 w/o all* 30.74 ± 1.14 SEM %) (Fig. 5C-D). For reprogramming DFs, the N2/Neurobasal/DMEM base medium resulted in a higher TAU purity and TH efficiency (Fig. 5D-E). The screen also revealed the importance of the canonical Wnt signaling activation molecule CHIR99021 as removing it decreased TH efficiency in all three base media *NDiff w/o CHIR99021* 2.01 ± 3.08 SEM %, *B27 w/o CHIR99021* 1.79 ± 0.49 SEM %, *N2 w/o CHIR99021* 5.17 ± 1.49 SEM % (Fig. 5E). Removing SMADi or VPA had the opposite effect and significantly increased the TH efficiency in N2/Neurobasal/DMEM (*N2 w/o SMADi* 19.98 ± 2.16 SEM %, *N2 w/o VPA* 20.41 ± 4.72 SEM %) (Fig. 5E). Based on these observations and the total TH counts (Table S8), we concluded that the N2/Neurobasal/DMEM base medium and the removal of VPA (*N2 w/o VPA*) from the small molecule mix significantly increased TH efficiency compared to the standard NDiff medium (*N2 w/o VPA* 20.41 ± 4.72 SEM %, NDiff standard 9.13 ± 2.69 SEM %) with high TAU purity and yield (Fig. 5C-E). We also measured DA release after KCl stimulation using GRAB_DA2M_ sniffer cells of all these conditions, but DA levels were below the detection level (results not shown). To assess whether the resulting TH^+^ neurons generated in the *N2 w/o VPA* expressed additional DA markers, we stained the iDANs (D21) for DA markers DDC, GIRK2 and aldehyde dehydrogenase 1A1 (ALDH1A1) and compared to standard NDiff (Fig. 5F-G). In addition, TH^+^ iDANs in *N2 w/o VPA* medium co-expressed the synaptic marker Synaptophysin (Fig. 5G). We observed more cells co-expressing DDC, GIRK2 or ALDH1A1 with TH in the *N2 w/o VPA* than standard NDiff.

## Discussion

In this study, we show that media composition and addition of small molecules are major determinants of TH reprogramming efficiency and that optimal conditions for direct conversion differ between starting cell types. By systematically comparing base media and small molecule combinations, we found that human GPCs and DFs benefit from distinct culture conditions during DA neuronal conversion.

Low reprogramming efficiency remains one of the major bottlenecks of direct reprogramming and its applications in disease modelling, drug screening and regenerative medicine. To date, significant efforts to improve efficiency have focused on TF combinations, vector design and expression systems^20,22,26,34^. In the initial development of direct neuronal reprogramming, multiple studies explored the addition of small molecules to improve the reprogramming and established that inhibiting the TGFβ and BMP pathway, activating the Wnt pathway as well as inhibiting histone deacetylation have a positive effect^9–11,21,35,36^. Many subsequent protocols developed for reprogramming GPCs, astrocytes and fibroblasts into iNs have since routinely used the same small molecule combinations at similar concentration regardless of starting cell type, base media and desired neuronal subtype^9,12,18,20,25,26,31,37^. We therefore sought to re-explore the influence of these now widely used base media and small molecules combinations specifically on DA reprogramming efficiency from two distinct starting human cell types.

We therefore systematically analyzed three base media; NDiff 227 that is a defined serum free medium supplemented with B27 and N2, Neurobasal supplemented with B27, and a 1:1 mix of DMEM/F12 and Neurobasal supplemented with N2. These media have been commonly used by us and others to reprogram fibroblasts and are also often used in neuronal differentiation^7,9,26,33,38–41^. Our results revealed starting cell-specific effects of the different base media. For GPCs, the highest TAU purities and TH efficiencies were achieved in B27/Neurobasal medium, while DFs reprogrammed better in the N2/DMEM/Neurobasal medium. One possible explanation is that the base media requirements reflect differences in developmental origin and reprogramming trajectory of the two starting cell types. GPCs share developmental lineage with neurons^42^, and has been reported to reprogram fast with TH^+^ cells being detectable as early as 7 days after induction^19^. In contrast, adult DFs must overcome a greater lineage barrier and may therefore require a base medium that better support their slower and more extensive lineage conversion. Therefore, the optimal base medium would not depend on the final neuronal subtype, but rather on the starting cell identity which is supported by our data.

Additional support for this came from the small molecule drop-out experiments. For GPCs reprogrammed in B27/Neurobasal, we found that the small molecules we usually include during neuronal conversion modulating SMAD and Wnt signaling and histone deacetylases were not needed. In fact, the removal of these molecules significantly improved DA reprogramming efficiency. This indicates that developmental pathway modulation is not required during ALNRi reprogramming and may even negatively affect DA reprogramming. Previous in vitro studies converting related cells such as astrocytes or pericytes have used small molecules such as CHIR99021, SB431542, LDN193189, Noggin and Dorsomorphin to improve neuronal reprogramming^24,43,44^. By contrast, in vivo studies reprogramming GPCs, astrocytes and astroglia suggest that the tissue provides sufficient support^3,24,45^. In line with this, our findings suggest that B27/Neurobasal with growth factors provide a sufficiently supportive in vitro environment for reprogramming GPCs, without the need for additional small molecules.

The iDANs generated in this simplified medium co-expressed DA markers and displayed functional properties similar to iDANs generated under standard NDiff plus small molecule conditions. However, the iDANs did not demonstrate spontaneous pacemaker-like firing, which is characteristic of mature midbrain DA neurons. This likely reflects the relatively early time point examined here (35 days), as electrophysiological properties develop progressively during reprogramming. Even so, the increased efficiency and total number of TH^+^ cells led to increased KCl-evoked DA release, which was detectable using the GRAB_DA2M_ sniffer cell assay already after 21 days of reprogramming. However, GPCs reprogrammed in *B27 w/o all* also had more proliferative cells (Ki67^+^) and showed higher expression of glial genes in our bulk RNA sequencing data. When non-reprogrammed GPCs were cultured in *B27 w/o all* or standard NDiff medium, we found that the GPCs proliferated more in *B27 w/o all*, indicating that the glial signatures detected in our sequencing data likely reflect continued proliferation of unconverted GPCs rather than a lack of neuronal maturation in the converted cells.

In contrast to GPCs, the DFs required some of the small molecules for efficient reprogramming into iDANs. In particular, we found that removing CHIR99021 from the small molecule cocktail had a negative effect on the TH efficiency while removing the histone deacetylase (HDAC) inhibitor, VPA or SMAD inhibition improved TH efficiency during ALNRi + LOF conversion. CHIR99021 is a canonical Wnt signaling activator and is used as a dose-dependent patterning factor in stem cell differentiation protocols, where relatively low concentrations specify the midbrain and higher concentrations caudalize towards the hindbrain^39,46^. In direct reprogramming protocols, CHIR99021 is commonly included to enhance conversion efficiency and is frequently used at high concentrations regardless of desired neuronal subtype^9,11,13^. Our findings support that CHIR99021 can improve neuronal conversion but also suggest that its effect depends on starting cell type and media context. In addition, CHIR99021 was tested only as a drop-out molecule. However, it would be interesting to titrate CHIR99021 during iDAN reprogramming to determine whether optimal conversion requires concentrations similar to those used in stem cell differentiation protocols^39,46^.The HDAC inhibitor, VPA is also commonly included to improve direct reprogramming protocols by modulating chromatin and lowering the epigenetic barrier to reprogramming. However, similar to our results, many studies also achieve successful reprogramming without VPA^9,13,34,37^. Overall, these differences in which small molecules are needed for reprogramming GPCs and DFs into iDANs highlight the importance of tailoring the culture conditions to the specific cells and reprogramming protocols used for optimal outcome. Thus, this screening-based strategy could be applied to optimize direct conversion across different starting cell types and target neuronal subtypes (interneuron, medium spiny neurons, motor neuron, cholinergic etc.^10,25,30–33^ than those used here. However, optimizing media composition alone could not completely overcome the relatively low efficiency of DA specification, as TH^+^ cells still represented only a subset of the reprogrammed iN population. This suggests that additional factors limit conversion efficiency. A likely technical limitation is the reprogramming strategy itself, which here relies on simultaneous transduction of multiple lentiviral vectors (4 and 8 individual vectors for GPCs and DFs respectively), resulting in heterogeneous transgene transduction across the culture. Consistent with this, we estimated that 32.33 ± 2.13 SEM % of the cultures expressed Ascl1, Lmx1a and Nurr1 (indicating complete delivery of the 4 factor reprogramming cassette). This technical limitation likely limits the maximum achievable reprogramming efficiency. We therefore expect that combining an optimized media composition with next-generation vector design, factor combinations, or delivery strategies would further increase DA reprogramming efficiency.

In conclusion, our results show that media composition is a major contributor to successful direct DA reprogramming, and that the media and small molecule requirements differ between human GPCs and DFs. We find that a B27 based medium w/o the small molecule cocktail significantly increased the reprogramming efficiency of GPCs into iDANs, whereas DFs in N2 based medium still depend on some of the small molecules but that removing VPA significantly improved reprogramming into iDANs. These findings suggest that routine use of neuronal conversion medium is not always optimal and should be optimized for each starting and target cell type.

## Methods

### Cell lines

For this study human embryonic stem cell (hESC) lines HS1001 (Kie055-A) and RC17 (Roslin Cells, hPSCreg RCe021-A) were used for generation of GPCs. In addition, four human dermal fibroblast lines (DFs) from the Parkinson’s Disease Research Clinic at Hohn van Geest Centre for Brain Repair (Cambridge, UK) were used. These fibroblast lines were obtained with subjects consent according to the declaration of Helsinki with local ethical approval at University of Cambridge, REC 09/H0311/88. Biopsies were sampled according to ^26^. One of the fibroblast lines (C11), was reprogrammed to human induced pluripotent stem cells (hiPSCs) as described in ^18^ and used to generate GPCs.

### Fibroblast culture

DFs were cultured in fibroblast medium consisting of DMEM GlutaMAX, 10% fetal bovine serum (FBS), and 1% penicillin-streptomycin in uncoated T175 flasks at 37 °C and 5% CO2. A complete media change was performed every 2–3 days. At 90% confluency, cells were expanded or replated for reprogramming experiments with 0.05% Trypsin-EDTA diluted in DPBS.

### Human glia progenitor (GPC) culture

GPCs were generated from hESCs and hiPSCs. These were maintained on plates coated with LN521 (BioLamina, 0.5 µg/cm^2^) in StemMACS iPS-Brew XF medium. Cells were passaged with 0.5 mM EDTA twice before starting differentiation into GPCs as previously described in ^20^ before being cryopreserved. Cryopreserved GPCs were thawed and cultured on tissue culture 6 well plates (Corning) coated with poly-L-ornithine (100 µg/mL) and laminin (5 µg/mL) and cultured in glial medium consisting of DMEM/F12 medium, B27 supplement (1:50), N1 supplement (1:100), MEM NEAA (1:100), Antibiotic-antimycotic (1:200) supplemented with T3 (60 ng/mL), biotin (100 ng/mL), recombinant human NT3 protein (10 ng/mL), db-cAMP (1 µM), recombinant human PDGF-AA protein (10 ng/mL) and recombinant human IGF-I (10 ng/mL).

### Fluorescence-activated cell sorting (FACS) analysis

To evaluate the generated human stem cell derived GPCs, FACS analysis was performed 14–35 days after thawing. Cells were dissociated with Accutase for 8 min at 37 °C and were resuspended in FACS buffer (PBS, 2 mM EDTA, 0.5% BSA Fraction V, 0.05% Phenol red) to a final concentration of 1 million cells/mL. Following dissociation, cells were incubated with fluorochrome-labeled antibodies (APC anti-human CD133/1, 1:40; FITC anti human SSEA-4, 1:40; PE anti-human CD140a, 1:10; APC anti-human CD44, 1:50,000) for 15 min in at 4 °C. After incubation, cells were washed with FACS Buffer and spun down for 10 min at 200 xg and samples were resuspended in DMEM/F12 with DNase and transferred to a 5 mL polystyrene tubes with 35 mm cell-strainer caps. Propidium iodide (PI, 1:100) was added to the samples for excluding dead cells in the analysis. Samples were analyzed on a FACSAria III Sorter (BD Biosciences), and 10,000 live events were recorded and analyzed. Final analysis of the FACS data was then performed in FlowJo 10.10 (Table S9).

### Lentivirus production

Third generation lentiviral vectors used for reprogramming were produced as previously described in Georgievska et al. and Zufferey et al.^47,48^. In brief, HEK293T cells were transfected with plasmid of interest along with three helper packaging plasmids, pMDL, psRev and envelope plasmid pMD2G using transfection reagent polyethyleneimine (PEI). The virus containing supernatant was collected 45 h after transfection, filtered using a 0.45 μm filter before ultracentrifugation at 25,000 xg at 4 °C for 1.5 h. The lentivirus was then resuspended in DPBS without calcium and magnesium and stored at -80 °C until used for experiments. To determine the titers of produced virus HEK293T cells were transduced with serial dilutions of the virus and a reference virus with titer confirmed by FACS. Transduced cells were collected and titer was determined by assessing WPRE expression in produced virus compared to reference virus by performing qPCR. Titers ranged between 5 × 10^8^ and 6 × 10^9^.

### Direct neuronal reprogramming

For 2D reprogramming cells were seeded onto plates sequentially coated overnight at 37 °C and 5% CO2 with poly-L-ornithine (15 µg/mL), laminin (5 µg/mL), and fibronectin (5 µg/mL). For reprogramming of DFs 25,000 cells/cm^2^ were plated and for GPCs 50,000 cells/cm^2^.

On the day of seeding DFs were transduced with a total of 8 lentiviral vectors, 6 containing single transgenes under PGK promoter (*Ascl1*, *Nr4A2 (Nurr1)*, *Lmx1A*, *Lmx1B*, *FoxA2* and *Otx2*) and 2 containing short hairpin RNAs targeting *REST* (ALNRi + LOF), an MOI of 5 per construct was used. The GPCs were instead transduced with 3 doxycycline-regulated lentiviral vectors expressing 3 transgenes (*Ascl1*, *Nr4A2 (Nurr1)* and *Lmx1A*, and 2 *REST* targeting shRNAs (ALNRi)) in addition to a Tet-On trans-activator at MOI of 1–2 per vector. The following day, a full media change was performed with fibroblast or glial medium depending on cell type. For reprogramming of GPCs doxycycline (2 µg/mL) was added to the media from day 1 onwards to activate expression of transgenes. On day 3 after seeding and transduction, culture media was exchanged to reprogramming media.

In the dropout experiment three base media were tested for the reprogramming: NDiff medium (NDiff 227 Medium with 1% penicillin-streptomycin), B27 medium (Neurobasal Medium supplemented with B27 without Vitamin A 1:50, MEM NEAA 1:100, L-Glutamine 1:100, penicillin-streptomycin 1:500), and N2 medium (50/50 mix of Neurobasal medium and DMEM/F12 supplemented with N2 supplement 1:100, MEM NEAA 1:100, L-Glutamine 1:100, penicillin-streptomycin 1:500). The media were supplemented with small molecules and growth factors (Table 1). From day 3 onwards half the media was exchanged with fresh media every 2–3 days.

**Table 1:** List of small molecules and growth factors used in each reprogramming media for drop out experiments, from day 3–9 for GPCs and day 3–16 for hDFs.

	Standard	w/o SMADi	w/o VPA	w/o CHIR99021	w/o all
SB431542 (10 µM)	X		X	X	
Noggin (50 ng/mL)	X		X	X	
LDN1931189 (0.5 µM)	X		X	X	
CHIR99021 (2 µM)	X	X	X		
VPA (1 mM)	X	X		X	
LM22A4 (2 µM)	X	X	X	X	X
GDNF (2 ng/mL)	X	X	X	X	X
NT3 (10 ng/mL)	X	X	X	X	X
db-cAMP (0.5 mM)	X	X	X	X	X

For reprogramming of GPCs Dual SMAD inhibition (SMADi), CHIR99021 and Valproic Acid (VPA) were removed on day 9 and media was just supplemented with LM22A4, GDNF, NT3 and db-cAMP (Fig. 1D top) and for reprogramming of DFs Dual SMADi, CHIR99021 and VPA was removed on day 16 (Fig. 1D bottom). Cells were kept for 14–21 days until collected for further analysis.

In final characterization experiments B27 base medium w/o all condition was used for GPC reprogramming from day 3 to end of experiment. As a non-DA reprogramming control, a polycistronic vector containing transgenes for *Ascl1*, *Brn2* and 2 *REST* targeting shRNAs (pB.pA) known to give rise to mostly GABAergic induced neurons was used^15,26^. For reprogramming of DFs N2 base medium w/o VPA condition was used from day 3–16, from day 16 onwards media was only supplemented with LM22A4, GDNF, NT3 and db-cAMP.

### Dopamine release

At the end of experiment before fixation, cells were stimulated for DA release with 150 mM KCl for 5 min at 37 °C and 5% CO2. After stimulation, media was collected and stored at -80 °C until analysis.

To assess DA levels, engineered HEK-293T Flp-In T-Rex cell line expressing fluorescent G protein-coupled receptor-based DA sensor GRAB_DA2M_ were used^49,50^. These cells were cultured in DMEM supplemented with 10% FBS, blasticidin (15 µg/mL) and Hygromycin B (200 µg/mL) and passaged twice before use in experiments. 24 h before measurement, cells were seeded in imaging plates (96-well Phenoplate, Revvity) coated with poly-L-ornithine. Tetracycline (1 µg/mL) was added to induce the expression of the GRAB_DA2M_ sensor. Imaging was performed using CV8000 (Yokogawa) microscope with a 10x air objective. Three images were taken per well: baseline fluorescence (F_0_), image after the addition of the sample (F_sample_), and a maximum fluorescence response after the addition of 10 µM DA solution (F_max_). Mean fluorescence intensity was extracted for each image using CellPathfinder (Yokogawa) and the response was reported as %max = (F_sample_-F_0_)/(F_max_-F_0_). Statistical analysis was performed in Prism (GraphPad).

### Whole-cell patch clamp in vitro

Electrophysiological recordings were performed on iDANs reprogrammed from GPCs using *B27 w/o all* media after 35 days of reprogramming. Coverslips with cells were transferred to recording chamber filled with artificial cerebrospinal fluid (aCSF) containing the following: 119 mM NaCl, 26 mM NaHCO_3_, 2.5 mM KCl, 1.25 mM NaH_2_PO_4_, 25 mM glucose, 2.5 mM CaCl_2_, and 1.3 MgSO_4_ with pH adjusted to 7.4 and an osmolarity of 307 mOsm. The aCSF solution was gassed with 95% O_2_ and 5% CO_2_ and perfused through recording chamber with a peristaltic pump while the chamber was kept at 34 °C using integrated heating system. The recordings were performed using an EPC-10 amplifier (HEKA Elektronik, Warner Instruments) controlled with PatchMaster software (HEKA Elektronik, Warner Instruments) with signals acquired at a 10 kHz sampling rate. Patch pipettes with pipette resistance of 3–7 MΩ were used and filled with intracellular solution consisting of 122.5 mM K-gluconate, 12.5 mM KCl, 10 mM HEPES, 8 mM NaCl, 2 mM MgATP, 0.3 mM Na_2_GTP adjusted to pH 7.2–7.3 and 296 mOsm. On day of recording, biocytin was added to intracellular solution to allow for visualization of cells and identification of recorded cells after recording.

After successful seal and break-in, cells were recorded in current clamp, resting membrane potential (RMP) was recorded immediately after break-in using 0 pA holding current. For further recording, holding current was adjusted if needed to keep membrane potential approximately − 70 mV. To evoke action potentials (APs), current was injected increasing 5 pA for each step (500 ms). Measurements for inward sodium and outward potassium were done in voltage clamp holding cells at -70 mV using depolarizing steps (500 ms) of increasing 10 mV. Data analysis was performed in Igor Pro 8, visualization and descriptive statistics were generated using GraphPad (Prism 10).

### Immunocytochemistry and microscopy

Cells were fixed with 4% paraformaldehyde for 10 min at room temperature (RT) and washed three times with DPBS without calcium and magnesium before staining. Cells were first incubated in blocking solution (0.1% Triton X-100 and 5% serum of same species as secondary antibodies in DPBS) for 1 h at RT. Following blocking, cells were incubated with primary antibodies diluted in blocking solution (see Table S10) overnight at 4 °C. The following day the cells were washed three times with DPBS before incubated with secondary antibodies (1:200, Jackson ImmunoResearch Laboratories) and DAPI (1:1,000) diluted in blocking solution for 1,5 h at RT. Cells were washed three times with DPBS before imaging. A Leica TCS SP8 confocal laser scanning microscope or a Leica DM16000B wide field inverted microscope with Leica LAS X software was used to capture fluorescence images.

To estimate co-delivery and expression of reprogramming factors, five images per well *were taken at random* and quantifications were performed manually using Fiji (Image J). All cells were counted based on DAPI staining and then positive cells for Ascl1, Nurr1 and Lmx1a were quantified. Lastly, positive cells for all three markers were quantified.

### High-Content Imaging Analysis

To perform quantifications, the Operetta CLS High-Content Analysis system (Revvity) was used, and images were acquired from 83 to 197 fields per well at 20x magnification in two planes with identical acquisition settings for each experiment. Analysis was performed in the Harmony software and maximum-intensity projection of the two planes were used for the analysis. The sliding parabola method was applied for background correction. Next nuclei segmentation was done based on DAPI staining and additional filtering by size, roundness and intensity was done to exclude cell debris, segmentation artifacts and correct for overlapping nuclei. Following selection of true nuclei total number of cells positive for TH and TAU were quantified and compared.

$$TAU\; yield (\%) = Number\; TAU \;positive \;cells / Number \;of \;seeded \;cells \times 100.$$

$$TAU \;purity (\%) = Number\; TAU \;positive \;cells / Number \;of \;nuclei \;detected \times 100.$$

$$TH \;efficiency (\%) = Number \;TH\; positive \;cells / Number \;TAU \;positive \;cells \times 100.$$

### Bulk RNA sequencing

For bulk RNA sequencing (Bulk RNA-seq) analysis, RNA was extracted using the RNeasy Mini KIT (Qiagen) according to the manufacturer’s instructions. Sequencing libraries were prepared using TruSeq Stranded mRNA Library Prep Kit (Illumina) according to the manufacturer’s instructions. Libraries were sequenced on Illumina NovaSeq 6000 with paired-end 150 bp reads. Raw sequencing reads were trimmed for adapter sequences and low-quality bases using Cutadapt (version 4.9). Trimmed reads were aligned to the human reference genome (GRCh38/hg38) using STAR (via the nf-core/rnaseq v3.17.0 pipeline). Gene-level counts were quantified using Salmon in alignment-based mode and merged across all samples into a single count matrix using tximport.

Gene-level counts and TPMs were analyzed in R. Lowly expressed genes were filtered by requiring at least 10 counts in at least 3 samples. Differential expression was performed in DESeq2, with Wald statistics from pairwise comparisons and apeglm shrinkage of log2 fold changes. Genes with adjusted *P* < 0.05 and absolute shrunken log2 fold change > 1 were considered significant. Variance-stabilized data were used for PCA. MA plots were generated in ggplot2 from DESeq2 results tables using the log10-transformed mean normalized expression for each gene (log10(baseMean + 1)) on the x-axis and apeglm-shrunken log2 fold change on the y-axis. GO Biological Process over-representation analysis and preranked GSEA were performed in clusterProfiler after mapping genes to Entrez identifiers with org.Hs.eg.db. Curated marker heatmaps were generated from log2(TPM + 1)-transformed, row-scaled TPM values. Analyses were performed with 3 samples per condition.

## Supplementary Information

Below is the link to the electronic supplementary material.

Supplementary Material 1

## Data Availability

Sequencing data generated in this study have been deposited in Gene Expression Omnibus (GEO) and are accessible under accession number GSE333803.
