# Supplementary material for: Distinct media requirements for dopaminergic neuron reprogramming from human glia and fibroblasts
Source: Sci Rep. 2026 Aug 17;16:25696. doi: 10.1038/s41598-026-66620-z (PMC13483191; doi:10.1038/s41598-026-66620-z)
Supplement: Supplementary file 1 [file 41598_2026_66620_MOESM1_ESM.docx]

**Supplementary information**

**Distinct media requirements for dopaminergic neuron reprogramming from human glia and fibroblasts**

Kerstin Laurin^1^, Janko Kajtez^2^, Jenny Wickham^1^, Malin Parmar^1^ & Mette Habekost^1,3,4,*^

^1^Developmental and Regenerative Neurobiology, Lund Stem Cell Center, Department of Experimental Medical Science, Faculty of Medicine, Lund University, Sweden.

^2^Novo Nordisk Foundation Center for Stem Cell Medicine (reNEW), Department of Biomedical Sciences,

Faculty of Health and Medical Sciences, University of Copenhagen, Copenhagen, Denmark.

^3^Center for Translational Neuromedicine, University of Copenhagen Faculty of Health and Medical Sciences, 2200 Copenhagen N, Denmark.

^4^Present address: Department of Biotechnology and Biomedicine, DTU Bioengineering, Technical University of Denmark, Kgs. Lyngby 2800, Denmark.

**Supplementary Figures:**

Fig S1

Fig S2

**Supplementary Tables:**

Table S1

Table S2

Table S3

Table S4

Table S5

Table S6

Table S7

Table S8

Table S9

Table S10

Supplementary Figure S1. A) Representative ICC images of GPCs reprogrammed with ALNRi in B27 w/o all media for 14 days stained for MASH1 (Ascl1), NURR1, LMX1A and DAPI to assess transduction efficiency. Scale bar 50 μm. B) Quantifications of all cells positive for Ascl1, Nurr1, Lmx1A or all three (Ascl1 + Nurr1 + Lmx1A); mean ± sd. N= 3 replicates (GPC batches from 2 ESC lines and 1 iPSC line) and 5 images per replicate.

Supplementary Figure S2. Gene ontology analysis of differentially expressed genes comparing ALNRi-reprogrammed GPCs in B27 w/o all or NDiff medium at day 10 (D10) or D21.Dot size indicates gene count and color indicates adjusted p-value.

Supplementary Table S1 – Cell counts related to Figure 1

|  | **# DAPI (total counts)** | **# TAU (total counts)** | **# TH in TAU (total counts)** | **# Analyzed fields** | **N replicates** |
| --- | --- | --- | --- | --- | --- |
| GPCs | 35,381 (± 21,379 SEM) | 581 (± 339 SEM) | 1 (± 1 SEM) | 83 | 3 |
| ALNRi (NDiff) | 15,916 (± 3,146 SEM) | 6,696 (± 801 SEM) | 345 (± 95 SEM) | 83 | 3 |
| DFs | 16,063 (± 417 SEM) | 22 (± 2 SEM) | 1 (± 1 SEM) | 143 | 4 |
| ALNRi+LOF (NDiff) | 10,510 (± 490 SEM) | 4,015 (± 329 SEM) | 176 (± 24 SEM) | 143 | 4 |

Supplementary Table S2 - Cell counts related to Figure 2

| **Condition** | **# DAPI (total counts)** | **# TAU (total counts)** | **# TH in TAU (total counts)** | **# Analyzed fields** | **N replicates** |
| --- | --- | --- | --- | --- | --- |
| NDiff (Standard) with all molecules | 36,270 (± 15,639 SEM) | 10,946 (± 2,637 SEM) | 195 (± 134 SEM) | 143 | 3 |
| w/o SMADi (NDiff) | 33,987 (± 11,213 SEM) | 10,203 (± 988 SEM) | 239 (± 144 SEM) | 143 | 3 |
| w/o VPA (NDiff) | 33,421 (± 11,276 SEM) | 12,260 (± 3,236 SEM) | 349 (± 185 SEM) | 143 | 3 |
| w/o CHIR (NDiff) | 29,207 (± 9,976 SEM) | 9,514 (± 1,761 SEM) | 366 (± 215 SEM) | 143 | 3 |
| w/o all (NDiff) | 44,200 (± 11,195 SEM) | 13,508 (± 3,610 SEM) | 763 (± 376 SEM) | 143 | 3 |
| B27/Neurobasal with all molecules | 38,268 (± 15,593 SEM) | 15,571 (± 4,419 SEM) | 1,479 (± 725 SEM) | 143 | 3 |
| w/o SMADi (B27) | 39,752 (± 20,646 SEM) | 13,159 (± 4,913 SEM) | 1,131 (± 633 SEM) | 143 | 3 |
| w/o VPA (B27) | 45,236 (± 23,774 SEM) | 15,329 (± 5,875 SEM) | 2,257 (± 1,147 SEM) | 143 | 3 |
| w/o CHIR (B27) | 38,871 (± 17,132 SEM) | 15,110 (± 4,933 SEM) | 2,522 (± 1,391 SEM) | 143 | 3 |
| w/o all (B27) | 39,914 (± 16,870 SEM) | 15,674 (± 5,248 SEM) | 3,001 (± 1,325 SEM) | 143 | 3 |
| N2/DMEM/Neurobasal with all molecules | 44,176 (± 26,168 SEM) | 12,565 (± 4,912 SEM) | 897 (± 629 SEM) | 143 | 3 |
| w/o SMADi (N2) | 47,688 (± 24,169 SEM) | 13,109 (± 5,731 SEM) | 976 (± 670 SEM) | 143 | 3 |
| w/o VPA (N2) | 44,429 (± 24,477 SEM) | 13,935 (± 5,566 SEM) | 1,445 (± 783 SEM) | 143 | 3 |
| w/o CHIR (N2) | 33,361 (± 21,727 SEM) | 9,559 (± 4,942 SEM) | 1,626 (± 1,294 SEM) | 143 | 3 |
| w/o all (N2) | 48,871 (± 21,229 SEM) | 14,783 (± 5,347 SEM) | 1,937 (± 736 SEM) | 143 | 3 |

Supplementary Table S3 - Cell counts related to Figure 3

|  | **# DAPI (total counts)** | **# TAU (total counts)** | **# TH in TAU (total counts)** | **# Analyzed fields** | **N replicates** |
| --- | --- | --- | --- | --- | --- |
| GPC | 118,700 (± 12,829 SEM) | 885 (± 297 SEM) | 1 (± 1 SEM) | 142 - 147 | 9 |
| ALNRi NDiff | 19,630 (± 1,752 SEM) | 12,750 (± 1,384 SEM) | 127 (± 30 SEM) | 142 - 147 | 12 |
| ALNRi B27 w/o all | 25,526 (± 3,955 SEM) | 13,906 (± 2,031 SEM) | 4,775 (± 776 SEM) | 142 - 147 | 12 |
| pB.pA B27 w/o all | 51,315 (± 4,557 SEM) | 8,300 (± 2,301 SEM) corresponding to 16.92 (± 5.49 SEM) % purity | 291 (± 101 SEM) corresponding to 3.5 (± 0.96 SEM) % efficiency | 143 | 6 |
| B27 w/o all only | 72,569 (± 4,445 SEM) | 6,157 (± 2,796 SEM) corresponding to 7.7 (± 3.36 SEM) % purity | 57 (± 17 SEM)  Corresponding to 1.84 (± 0.51 SEM) % efficiency | 143 | 6 |

Supplementary Table S4 - Cell counts related to Figure 3

|  | **# DAPI**  **(total counts)** | **# NeuN**  **(total counts)** | **# TH**  **(total counts)** | **# Analyzed fields** | **N replicates** |
| --- | --- | --- | --- | --- | --- |
| GPC | 81,035 (± 3,289 SEM) | 358 (± 38 SEM) | 17 (± 3 SEM) | 101 | 3 |
| ALNRi  NDiff | 32,162 (± 5,836 SEM) | 6,313 (± 1,797 SEM) | 21 (± 8 SEM) | 101 | 3 |
| ALNRi  B27 w/o all | 36,401 (± 5,760 SEM) | 9,103 (± 1,957 SEM) | 1,773 (± 335 SEM) | 101 | 3 |

Supplementary Table S5 – Descriptive information from Patch Clamp experiments Figure 3

|  | **RMP**  **[mV]** | **Ri**  **[GΩ]** | **RMP**  **[mV]** | **Ri**  **[GΩ]** | **AP**  **threshold**  **[mV]** | **AP**  **amplitude**  **[mV]** | **AHP**  **[mV]** | **AP halfwidth**  **[ms]** |
| --- | --- | --- | --- | --- | --- | --- | --- | --- |
| Number of values | 9 | 9 | 6 | 6 | 6 | 6 | 6 | 6 |
| group | All cells | All cells | Only AP | Only AP | Only AP | Only AP | Only AP | Only AP |
| Minimum | -66.00 | 0.1667 | -54.00 | 0.1667 | -28.67 | 17.75 | -42.07 | 3.398 |
| 25% Percentile | -51.50 | 0.6861 | -50.25 | 0.5351 | -27.07 | 18.64 | -41.62 | 3.442 |
| Median | -34.00 | 0.9259 | -36.50 | 0.9732 | -19.61 | 26.43 | -36.48 | 4.091 |
| 75% Percentile | -28.00 | 1.640 | -28.00 | 1.932 | -17.25 | 30.10 | -32.25 | 5.737 |
| Maximum | -22.00 | 2.174 | -22.00 | 2.174 | -14.99 | 33.27 | -30.99 | 6.383 |
| Range | 44.00 | 2.007 | 32.00 | 2.007 | 13.68 | 15.52 | 11.08 | 2.985 |
| Mean | -39.22 | 1.083 | -38.00 | 1.133 | -21.24 | 25.31 | -36.69 | 4.490 |
| Std. Deviation | 14.38 | 0.6287 | 11.95 | 0.7504 | 5.262 | 5.996 | 4.524 | 1.204 |
| Std. Error of Mean | 4.792 | 0.2096 | 4.879 | 0.3063 | 2.148 | 2.448 | 1.847 | 0.4915 |

Supplementary Table S6 - Cell counts related to Figure 4

|  | **# DAPI**  **(total counts)** | **# Ki67**  **(total counts)** | **# TH**  **(total counts)** | **# Analyzed fields** | **N replicates** |
| --- | --- | --- | --- | --- | --- |
| ALNRi  NDiff | 27,215 (± 3,728 SEM) | 746 (± 89 SEM) | 41 (± 16 SEM) | 197 | 3 |
| ALNRi  B27 w/o all | 54,275 (± 1,029 SEM) | 3,691 (± 188 SEM) | 7,699 (± 195 SEM) | 197 | 3 |

Supplementary Table S7 - Cell counts related to Figure 4

|  | **# DAPI**  **(total counts)** | **# Ki67**  **(total counts)** | **# Analyzed fields** | **N replicates** |
| --- | --- | --- | --- | --- |
| GM | 126,012 (± 23,362 SEM) | 60,037 (± 23,027 SEM) | 144 | 3 |
| B27 w/o all | 64,689 (± 9,197 SEM) | 16,455 (± 4,079 SEM) | 144 | 3 |
| NDiff | 35,798 (± 1,758 SEM) | 3,369 (± 655 SEM) | 144 | 3 |

Supplementary Table S8 - Cell counts related to Figure 5

| **Condition** | **# DAPI (total counts)** | **# TAU (total counts)** | **# TH in TAU (total counts)** | **# Analyzed fields** | **N replicates** |
| --- | --- | --- | --- | --- | --- |
| NDiff (standard) with all molecules | 9,374 (± 1,543 SEM) | 4,632 (± 817 SEM) | 436 (± 184 SEM) | 143 | 3 |
| w/o SMADi (NDiff) | 6,614 (± 823 SEM) | 1,989 (± 590 SEM) | 248 (± 55 SEM) | 143 | 3 |
| w/o VPA (NDiff) | 6,850 (± 847 SEM) | 3,570 (± 512 SEM) | 628 (± 184 SEM) | 143 | 3 |
| w/o CHIR (NDiff) | 7,571 (± 1,036 SEM) | 3,155 (± 539 SEM) | 57 (±10 SEM) | 143 | 3 |
| w/o all (NDiff) | 7,195 (± 1,045 SEM) | 1,457 (± 539 SEM) | 105 (±29 SEM) | 143 | 3 |
| B27/Neurobasal with all molecules | 11,285 (± 2.318 SEM) | 4,013 (± 742 SEM) | 179 (± 55 SEM) | 143 | 3 |
| w/o SMADi (B27) | 9,133 (± 775 SEM) | 2,388 (± 378 SEM) | 319 (± 58 SEM) | 143 | 3 |
| w/o VPA (B27) | 9,711 (± 505 SEM) | 3,557 (± 325 SEM) | 314 (± 34 SEM) | 143 | 3 |
| w/o CHIR (B27) | 8,336 (± 3,222 SEM) | 2,954 (± 260 SEM) | 50 (± 11 SEM) | 143 | 3 |
| w/o all (B27) | 6,652 (± 621 SEM) | 1,158 (± 247 SEM) | 68 (± 23 SEM) | 143 | 3 |
| N2/DMEM/Neurobasal with all molecules | 6,820 (± 621 SEM) | 3,180 (± 489 SEM) | 452 (± 209 SEM) | 143 | 3 |
| w/o SMADi (N2) | 4,820 (± 738 SEM) | 2,383 (± 330 SEM) | 467 (± 45 SEM) | 143 | 3 |
| w/o VPA (N2) | 6,838 (± 695 SEM) | 3,654 (± 631 SEM) | 763 (± 259 SEM) | 143 | 3 |
| w/o CHIR (N2) | 6,999 (± 770 SEM) | 3,093 (± 308 SEM) | 161 (± 55 SEM) | 143 | 3 |
| w/o all (N2) | 4,916 (± 585 SEM) | 1,438 (± 151 SEM) | 144 (± 22 SEM) | 143 | 3 |

Supplementary Table S9 – FACS data of hGPC batches included in the study. Days in culture shows days of differentiation before cryopreservation + days post-thaw. If kept longer than >28 days post-thaw cells were passaged once before start of experiment.

| Cell line | Type of cells | hGPC batch ID | Days in culture | CD140^+^ / CD44^-^ (%) | CD140^+^ / CD44^+^ (%) | CD140^-^ / CD44^+^ (%) | SSEA4- / CD133^+^ (%) |
| --- | --- | --- | --- | --- | --- | --- | --- |
| RC17 | ESC | LU24 | D229+28 | 44.6 | 12.6 | 0.2 | 36.5 |
| HS1001 | ESC | LU27 | D224+28 | 70.0 | 22.2 | 0.5 | 63.3 |
| HS1001 | ESC | LU29 | D229+28 | 71.8 | 14.7 | 0.2 | 63.3 |
| RC17 | ESC | J4 | D226+28 | 52.3 | 4.2 | 0.4 | 26.2 |
| HS1001 | ESC | LU29 | D194+27 | 60.4 | 23.4 | 0.4 | 65.4 |
| RC17 | ESC | LU34 | D190+27 | 65.2 | 25.3 | 0.2 | 63.3 |
| C11 | iPSC | KL16 | D181 | 62.8 | 0.8 | 0 | 48 |
| HS1001 | ESC | LU29 | D194+14 | 39.3 | 20.2 | 2.8 | 35 |
| RC17 | ESC | LU34 | D190+14 | 55.6 | 13.5 | 1.8 | 43.8 |
| C11 | iPSC | KL16 | D195+14 | 53.4 | 11.7 | 0.4 | 51.5 |
| RC17 | ESC | LU28 | D230+41 | 69.9 | 12.7 | 0.4 | 40 |

Supplementary Table S10 – List of primary antibodies used in this study.

| Marker | Specificity | Source (cat. no.) | Dilution |
| --- | --- | --- | --- |
| ALDH1A1 | Goat | R&D Systems (AF5869) | 1:500 |
| COL1A1 | Sheep | R&D Systems (AF6220) | 1:500 |
| DDC | Rabbit | Millipore (AB1569) | 1:500 |
| GIRK2 | Rabbit | Alamone (APC 006) | 1:200 |
| Ki67 | Rabbit | Abcam (ab16667) | 1:500 |
| LMX1A | Goat | Santa Cruz (sc54274) | 1:200 |
| MASH1 (Ascl1) | Rabbit | Abcam (ab38556) | 1:500 |
| NeuN | Rabbit | Millipore (ABN78) | 1:500 |
| NURR1 | Mouse | Abcam (ab41917) | 1:500 |
| PDGFRα | Rabbit | Cell Signaling (5241S) | 1:300 |
| STEM123 (GFAP) | Mouse | Takara (Y40420) | 1:500 |
| Synapsin 1 | Rabbit | Cell Signaling (D12G5) | 1:300 |
| Synaptophysin | Mouse | Millipore (MAB329) | 1:200 |
| TAU (HT7) | Mouse | Thermo Fisher (MN1000) | 1:500 |
| TH | Rabbit | Millipore (AB152) | 1:1000 |
| TH | Sheep | Millipore (AB1542) | 1:500 |
